# Lineage-restricted dependency on an oncofetal *SNHG29*-IGF2BP1 RNA axis in acute megakaryoblastic leukemia

**DOI:** 10.1038/s41375-026-03040-y

**Published:** 2026-06-30

**Authors:** Robert Winkler, Bruno Griesler, Wolfgang Sippl, Reinier A. Boon, Stefanie Dimmeler, Ilka Wittig, Marie-Laure Yaspo, Stefan Hüttelmaier, Raj Bhayadia, Dirk Heckl, Jan-Henning Klusmann

**Affiliations:** 1 Department of Pediatrics, Goethe University Frankfurt, Frankfurt am Main, Germany; 2 Frankfurt Cancer Institute, Frankfurt/Main, Germany; 3 German Cancer Consortium (DKTK), German Cancer Research Center (DKFZ), Partner site, Frankfurt/Mainz, Germany; 4 Institute of Molecular Medicine, Martin Luther University Halle-Wittenberg, Halle, Germany; 5 University Clinic and Outpatient Clinic for Internal Medicine IV, University Medicine Halle (Saale), Halle (Saale), Germany; 6 Institute for Cardiovascular Regeneration, Centre for Molecular Medicine, Goethe University, Frankfurt Am Main, Germany; 7 German Centre for Cardiovascular Research, Partner Site Frankfurt Rhein/Main, Frankfurt, Germany; 8 Department of Physiology, Amsterdam University Medical Centers, Vrije Universiteit Amsterdam, Amsterdam, the Netherlands; 9 Amsterdam Cardiovascular Sciences, Amsterdam, the Netherlands; 10 Max Planck Institute for Molecular Genetics, Berlin, Germany; 11 School of Health Sciences and Technology, UPES, Dehradun, Uttarakhand India; 12 Institute for Experimental Pediatric Hematology and Oncology, Goethe University Frankfurt, Frankfurt am Main, Germany

**Keywords:** Acute myeloid leukaemia, Haematopoiesis, Targeted therapies

## Abstract

Acute megakaryoblastic leukemia (AMKL) is a rare, aggressive subtype of acute myeloid leukemia with developmental origins in early childhood. To uncover long noncoding RNAs (lncRNAs) sustaining this high-risk malignancy, we conducted CRISPR interference screens targeting lncRNAs overexpressed in primary AMKL samples. This analysis identified *SNHG29* as a previously unrecognized lineage-specific dependency, whose silencing profoundly impaired leukemic proliferation and clonogenic growth in vitro and reduced leukemic burden in vivo. RNA pulldown coupled with proteomic analysis revealed that *SNHG29* interacts with the oncofetal RNA-binding protein IGF2BP1, which is aberrantly expressed in AMKL. *SNHG29* was required to maintain expression of IGF2BP1 target transcripts, including MYC- and E2F-driven proliferative programs, thereby reinforcing fetal transcriptional programs essential for leukemic maintenance. Pharmacologic inhibition of IGF2BP1-RNA interactions with the small-molecule BTYNB induced potent and selective cytotoxicity in patient-derived AMKL models. Together, these findings uncover a developmental co-dependency between *SNHG29* and IGF2BP1 that defines a lineage-restricted oncogenic circuit and an actionable therapeutic vulnerability in AMKL.

## Introduction

Long non-coding RNAs (lncRNAs) are transcripts longer than 200 nucleotides that lack protein-coding potential [1, 2], although an increasing number have been shown to harbor small open reading frames encoding functional microproteins [3, 4]. In contrast to protein-coding genes, lncRNAs display highly specific expression patterns, often restricted to distinct cell lineages, developmental stages, or disease contexts, suggesting specialized biological roles [5, 6]. They act through multiple mechanisms—by scaffolding protein complexes [7], guiding chromatin modifiers [8], modulating mRNA stability [9], or sequestering microRNAs [10, 11]—to fine-tune transcriptional and post-transcriptional programs. Recent work has uncovered central roles for lncRNAs in hematopoiesis, where they control hematopoietic stem cell maintenance, lineage commitment, and terminal differentiation [12, 13]. Dysregulated lncRNA expression contributes to malignant transformation across hematologic neoplasms [14–16], including acute leukemias, underscoring their potential as therapeutic targets.

Acute megakaryoblastic leukemia (AMKL) is a rare and aggressive subtype of acute myeloid leukemia (AML) characterized by uncontrolled proliferation of megakaryoblasts. AMKL has developmental ties to early hematopoiesis and frequently arises in early childhood [17], particularly in the context of Down syndrome (ML-DS), which has a favorable outcome [18, 19]. In contrast, non–Down syndrome AMKL (non-DS-AMKL) is driven by high-risk fusion oncogenes such as *CBFA2T3::GLIS2*, *NUP98::KDM5A* or *RBM15::MKL1* [20, 21], and is associated with poor survival despite intensified chemotherapy [22, 23], highlighting the urgent need for innovative, molecularly guided therapies.

We hypothesized that the distinct transcriptional landscape of AMKL—shaped by developmental lineage programs and fusion-driven oncogenic signaling—creates dependencies on specific long non-coding RNAs that sustain leukemic identity and survival through post-transcriptional regulatory mechanisms. To test this hypothesis, we performed a high-throughput CRISPR interference (CRISPRi) screen targeting lncRNAs that are overexpressed in primary AMKL samples. This approach identified *SNHG29* as a selective and essential dependency. Using complementary genetic perturbation strategies, we established its functional relevance in vitro and in vivo, delineated its protein interactome, and uncovered a mechanistic link to the oncofetal RNA-binding protein (RBP) IGF2BP1. We further explored therapeutic disruption of this lncRNA–RBP axis. Together, our findings identify *SNHG29* as a critical lncRNA in AMKL and reveal a previously unrecognized *SNHG29*–IGF2BP1 regulatory axis that represents an actionable vulnerability in megakaryoblastic leukemia.

## Methods

### CRISPR library design, cloning and screening

We employed a custom CRISPRi library designed to target a comprehensive list of lncRNAs assembled from GENCODE v25(03/2016 release) [24], LNCipedia 4.0 (05/2016 release) [25], and NONCODE v4 (01/2014 release) [26], which was previously developed by our group [27]. The library comprises three to nine sgRNAs per target, tiling the 250 bp window immediately downstream of the annotated transcription start site. The library incorporated non-targeting controls against luciferase and positive depletion controls targeting essential genes such as *MYC*, *MYB*, and *RPL9*. Information on library cloning, cell culture and data processing are provided in the Supplementary Methods.

### RNA affinity purification

RNA affinity purification was performed based on a protocol previously described by Neumann et al. [28]. For each experimental condition, 20 million M-07e cells were washed and lysed in lysis buffer (Tris-HCl 50 mM, NaCl 150 mM, EDTA 1 mM, 1% NP-40, Roche c0mplete protease inhibitor). The resulting lysate was pre-cleared with streptavidin beads for 2 h at 4 °C, then incubated with 200 pmol of 2’O-Me-RNA antisense oligonucleotides for 1 h at 37 °C. To capture the RNA-protein complexes, we used streptavidin beads that had been pre-blocked with 0.2 mg/ml each of ytRNA (Thermo Fisher Scientific) and glycogen (Thermo Fisher Scientific). The beads were subjected to five washes in wash buffer (50 mM Tris-HCl pH8, 50 mM NaCl, 0.05% NP-40, Roche c0mplete Protease inhibitor), excluding protease inhibitors and NP-40 during the final wash step. Elution was performed by incubating the beads for 30 min at 37 °C in 50 µL of elution buffer (10 mM D-Biotin, 10 mM Tris-HCl, 50 mM NaCl). Eluates were analyzed by qRT-PCR and relative *SNHG29* enrichment calculated over input samples. SDS-PAGE was stained using the SilverQuest silver staining kit (Thermo Fisher Scientific).

Eluates were trypsinated and LC-MS/MS was performed on a Q Exactive Plus instrument (Thermo Fisher Scientific) equipped with an ultra-high performance liquid chromatography unit (Thermo Scientific Dionex Ultimate 3000). For data analysis using t-test and multiple testing correction MaxQuant 2.0.3.0 [29], Perseus 2.0.7.0 [30] and Excel (Microsoft Office 2016) were used. The UniProt [31] human reference proteome database (October 2022, 102557 entries) was used to identify peptides and proteins. Reverse identifications and common contaminants were removed. Missing values were replaced by background values from normal distribution. Proteins with Log_2_FoldChange > 4 and adjusted *P* < 0.01 in the *SNHG29*-ASO vs NT-ASO condition were considered for downstream analysis.

### Enhanced UV crosslinking and immunoprecipitation (eCLIP) analysis

eCLIP data for proteins showing significant binding in the RNA affinity purification was retrieved from the Encode consortium. Data consisted of 2 replicates in HepG2, K562 or both. K562 experiments were preferred where available. Preprocessing steps, including alignment to hg38 and peak calling using CLIPper, were previously carried out by the ENCODE consortium. Individual replicate peaks were mapped to Ensembl version 108 [32] transcripts using bedtools [33]. To orthogonally validate protein binding to *SNHG29*, specific criteria were applied: a peak had to be present on an *SNHG29* exon within 100 bp in both replicates, with a Log_2_ Fold Change >3 over size-matched input and a -Log_10_ adjusted *P* value > 4. PyGenomeTracks was used to visualize eCLIP peaks on the *SNHG29* transcript. Target genes were classified as significantly bound if a reproducible peak was found in the IDR peak set. Both intronic and exonic binding was considered. Bound genes were ranked by Log_2_ Fold Change. Gene sets were created after sorting by Log_2_ Fold Change to select 250 genes for gene set enrichment analysis (GSEA).

### RNA sequencing

Total RNA was extracted on days 3 (shRNA) or 7 (CRISPRi) post-transduction using the RNeasy Plus Mini Kit (Qiagen). rRNA-depleted libraries were sequenced on an Illumina NovaSeq 6000 (150 bp paired-end; Novogene). Reads were processed using the nf-core/rnaseq pipeline [34, 35], aligned to hg38 with STAR [36], and quantified with Salmon [37]. Differential expression analysis was performed using DESeq2 [38] for SNHG29 knockdown experiments and LIMMA [39] for patient data. Gene set enrichment analysis was carried out using fgsea [40]. Detailed processing steps and parameters are described in the Supplementary Methods.

### Statistical analysis

CRISPRi screen data were analyzed using MAGeCK [41]. Differential expression analysis was performed using DESeq2 [38] and LIMMA [39] for SNHG29 knockdown and patient data, respectively. Correlation coefficients were compared by two-tailed *t* test after Fisher z-transformation. SNHG29 enrichment in the RNA affinity purification was analyzed using a ratio t-test to appropriately model the proportional enrichment of the pulldown relative to the control. For all other experiments, two-tailed unpaired t-tests were used unless otherwise noted. Normal distribution and equal variance were assumed, consistent with standard practice for the given assay, but could not be formally tested given the small sample sizes of most experiments. Statistical analyses were performed in R and GraphPad Prism 9. Data are presented as mean ± s.e.m. with sample sizes indicated in figure legends. *P* < 0.05 was considered significant. Sample sizes were based on experimental feasibility and prior experience with similar assays. For colony counting, the experimentalist was blinded to the experimental condition. Otherwise, no blinding was performed, as experiments generated objective quantitative readouts.

### Animal studies

Animal experiments utilized immunodeficient mice (Regeneron Pharmaceuticals), which were maintained in a pathogen-free facility in individually ventilated cages with access to autoclaved food and water in a 12-h light/dark cycle. All animal procedures were approved by the Regierungspräsidium Darmstadt and conducted at Goethe University Frankfurt.

### Experimental model and study participant details

All human samples were collected with informed consent from participants or their legal guardians, under protocols approved by the ethics committee of the Martin Luther University Halle-Wittenberg. Primary acute myeloid leukemia (AML) samples were sourced from Goethe University Frankfurt, the AML-BFM Study Group (Essen, Germany), and the Department of Hematology, Hemostasis, Oncology, and Stem Cell Transplantation at Hannover Medical School.

Additional and detailed descriptions of procedures can be found in Supplementary Methods.

## Results

### Systematic investigation of AMKL lncRNAs using CRISPRi screens

**Fig. 1:**
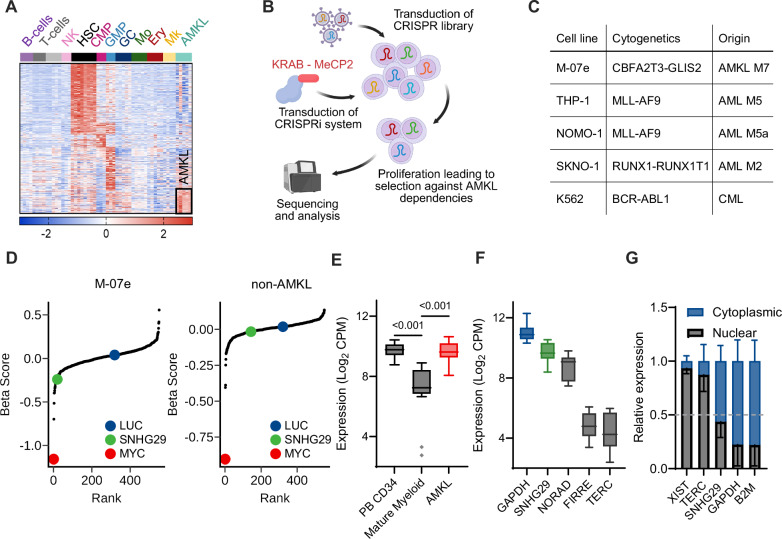
A CRISPRi screen identifies SNHG29 as a selective lncRNA dependency in AMKL. **A** LncRNA expression profiling in primary AMKL patient samples and a panel of normal hematopoietic cell populations. Differential analysis reveals a unique signature enriched in AMKL, which is highlighted by the black rectangle. Expression values are shown as row-wise z-scores. NK natural killer cell, HSC hematopoietic stem cell, CMP common myeloid progenitor, GMP granulocyte-monocyte progenitor, GC granulocyte, Mo monocyte, Ery erythroid precursor, Mk megakaryocyte, AMKL acute megakaryoblastic leukemia patient samples. **B** Schematic of the CRISPRi screening workflow designed to functionally interrogate the AMKL lncRNA signature. **C** Cell lines included in the screening, their cytogenetic characteristics, and disease of origin. **D** CRISPRi screen results showing gene essentiality scores in M-07e cells (left) and all other cell lines (right), calculated by MAGeCK MLE. **E** Expression levels of *SNHG29* in RNA sequencing datasets from peripheral blood mobilized CD34+ cells (PB CD34, *n* = 8), differentiated myeloid cells (*n* = 16, monocytes, late megakaryocytic and erythroid progenitors, granulocytes), and AMKL patient samples (*n* = 17). Data shown as LIMMA-voom transformed counts per million on Log_2_ scale. ****FDR < 0.0001 (LIMMA-voom). Box plots show medians, boxes, and whiskers according to the Tukey method. **F** Expression levels of *SNHG29* (pink), other lncRNAs (green), and protein-coding genes (gray) in AMKL patient samples (*n* = 17). Data shown as LIMMA-voom transformed counts per million on log_2_ scale. Box plots show medians, boxes, and whiskers according to the Tukey method. **G** *SNHG29* abundance in nuclear (gray) versus cytoplasmic (pink) fractions, quantified by qRT-PCR. Localization is compared to established nuclear (XIST, TERC) and cytoplasmic (GAPDH, B2M) control transcripts. Data are presented as percentage of total detected transcript in each fraction (*n* = 2, mean ± s.e.m).

To define the transcriptional landscape underlying AMKL, we performed comprehensive lncRNA expression profiling across FACS-sorted hematopoietic stem cells, progenitors, mature blood lineages, and pediatric AML blasts spanning multiple cytogenetic subtypes. This analysis revealed a distinct lncRNA signature selectively enriched in AMKL blasts relative to all other hematopoietic populations and AML subtypes (Fig. 1A) [42].

To assess the functional relevance of these lncRNAs, we performed a high-throughput CRISPR interference (CRISPRi, dcas9.KRAB.MeCP2) [43] screen targeting 548 noncoding RNA genes (Fig. 1B). The screen was carried out in the AMKL-derived M-07e line, which harbors a *CBFA2T3::GLIS2* fusion, alongside a reference panel of AML cell lines representing alternative subtypes (Fig. 1C). Among the top-scoring hits, *SNHG29* emerged as a strong and selective dependency in AMKL, with its knockdown markedly reducing M-07e viability (Fig. 1D), while having minimal impact on non-AMKL cell lines. Consistently, *SNHG29* was highly expressed in AMKL patient samples and downregulated during normal hematopoietic differentiation (Fig. 1E), is abundant relative to other lncRNAs (Fig. 1F) and exhibits both nuclear and cytoplasmic localization (Fig. 1G). Together, these results identify *SNHG29* as a candidate lineage-specific dependency lncRNA in AMKL.

### SNHG29 is a lineage-specific dependency lncRNA in AMKL

**Fig. 2:**
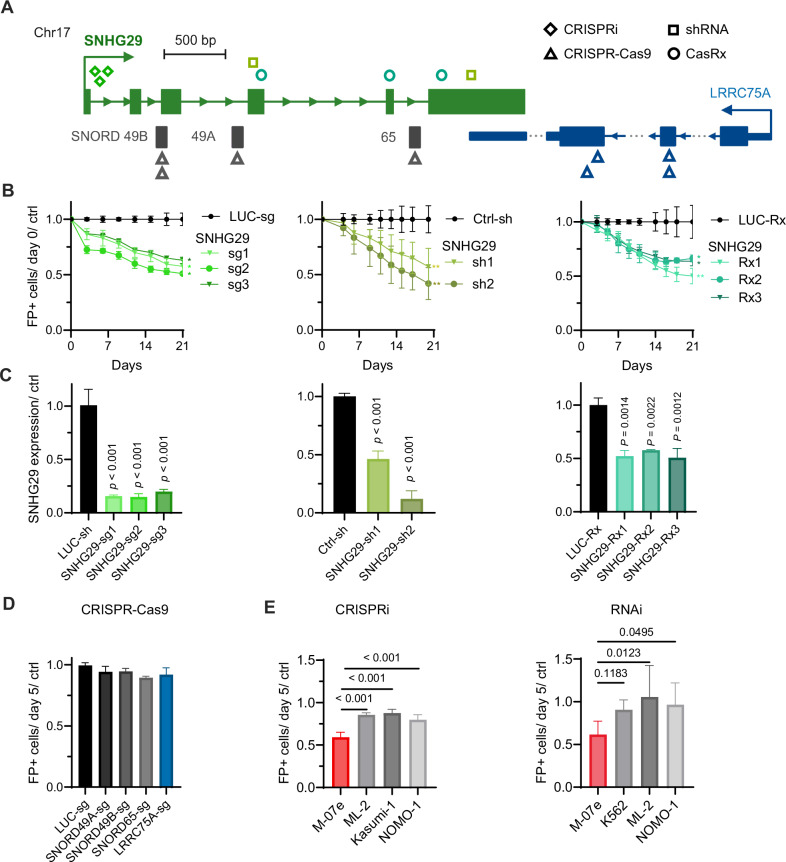
Orthogonal loss-of-function approaches confirm SNHG29 as an AMKL-selective dependency. **A** Schematic representation of the *SNHG29* genomic locus on chromosome 17. Arrow indicates transcriptional orientation. Boxes and connecting lines represent exons and introns of *SNHG29*, respectively. Symbols at the bottom indicate target sites for different perturbation strategies: CRISPRi (diamonds), shRNA (squares), CRISPR-CasRx (circles), and CRISPR-Cas9 (triangles). **B** Fluorescence-based proliferation assays in M-07e cells using different *SNHG29* knockdown strategies. Left: CRISPRi using three sgRNAs (*n* = 2). Middle: RNAi using two shRNAs (*n* = 5 for *SNHG29*-sh1, n = 4 for *SNHG29*-sh2). Right: CRISPR-CasRx using three sgRNAs (*n* = 3, except n = 2 for Rx3). Data normalized to day 0 and respective controls. (mean ± s.e.m; *P < 0.05, **P < 0.01, ***P < 0.001; two-tailed, unpaired *t* test) **C** *SNHG29* expression in M-07e cells via qRT-PCR. Left: CRISPRi knockdown (*n* = 3 for sg1 and sg2, n = 2 for sg3). Middle: RNAi knockdown (*n* = 5 for sh1, *n* = 4 for sh2). Right: CRISPR-CasRx knockdown (n = 2, mean ± s.e.m.). Data normalized to respective controls. (mean ± s.e.m.; *P < 0.05, **P < 0.01, ***P < 0.001; two-tailed, unpaired *t* test) **D** Cell depletion at the final timepoint of fluorescence-based proliferation assay in M-07e cells using CRISPR-Cas9 targeting indicated SNORD loci and LRRC75A. Data normalized to day 0 and a non-targeting control sgRNA (*n* = 3 biological replicates for LRRC75A, *n* = 2 for other conditions, mean ± s.e.m.). **E** Cell depletion at the final timepoint of competitive proliferation assays targeting *SNHG29* in different AML cell lines. Left: CRISPRi-mediated depletion at day 18 (*n* = 2 biological replicates, pooled data from 3 sgRNAs per cell line). Right: shRNA-mediated depletion at day 15 for M-07e cells and day 14 for other cell lines (*n* = 4 in M-07e, *n* = 2 for all other conditions, pooled data from 2 shRNAs per cell line). Data normalized to day 0 and controls (LUC-sg for CRISPRi, Ctrl-sh for RNAi). (mean ± s.e.m.; ***P < 0.001.; two-tailed, unpaired *t* test).

To validate the CRISPRi screen results, we employed complementary loss-of-function strategies including shRNA-mediated knockdown, RNA-targeting by CRISPR-Cas13d (RfxCas13d, CRISPR-CasRx), and CRISPR-Cas9-mediated genomic deletion (Fig. 2A). All perturbations confirmed that *SNHG29* is essential for AMKL cell proliferation (Fig. 2B). shRNA-mediated knockdown of *SNHG29* significantly impaired M-07e growth, with effects proportional to knockdown efficiency (Fig. 2C). CRISPR-CasRx also recapitulated these results.

*SNHG29* hosts several C/D box snoRNAs (SNORD49A, SNORD49B, SNORD65). To test whether these embedded elements mediate the observed phenotype, we targeted conserved C/D motifs using CRISPR-Cas9 as previously described [44]. In parallel, we noted that *SNHG29* knockdown modestly reduced expression of its neighboring coding gene, *LRRC75A* (Fig. S1A). However, knockout of *LRRC75A* or the embedded snoRNAs did not affect proliferation, even accounting for variation in sgRNA efficiency (Fig. 2D, Fig. S1B). These findings indicate that the functional dependency resides in the *SNHG29* transcript itself, rather than its hosted snoRNAs or neighboring gene.

We next extended these findings to additional AML models. Only AMKL cell line M-07e exhibited growth inhibition upon *SNHG29* depletion (Fig. 2E), whereas non-AMKL cell lines were unaffected, confirming the specificity of this dependency.

**Fig. 3:**
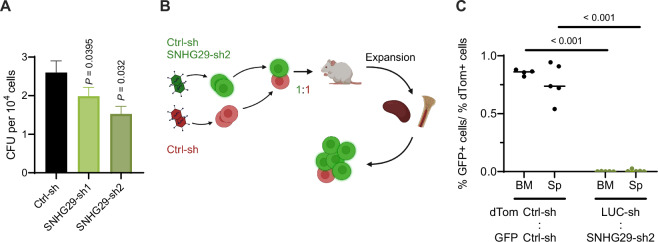
SNHG29 depletion impairs clonogenic capacity and in vivo propagation of AMKL patient-derived cells. **A** Colony forming assay in AMKL PDX cells following *SNHG29* knockdown (n = 3 biological replicates, mean ± s.e.m.; *P < 0.05, **P < 0.01) **B** Experimental setup for in vivo competition assays using AMKL PDX cells. **C** In vivo competition assay showing ratio of GFP to dTomato-labeled cells in bone marrow (BM) and spleen (Sp) of transplanted mice (*n* = 5 biological replicates, mean ± s.e.m.; ***P < 0.001, ****P < 0.0001; two-tailed, unpaired *t* test).

To test functional relevance in patient-derived material, we performed colony assays following *SNHG29* knockdown in primary AMKL blasts, which showed a significant reduction in colony-forming potential upon *SNHG29* depletion (Fig. 3A). In competitive xenotransplantation assays, PDX cells transduced with *SNHG29* shRNA or a control (*LUC* shRNA) were co-injected into immunodeficient mice at a 1:1 ratio (Fig. 3B). Endpoint analysis revealed that *SNHG29*-depleted cells were nearly eliminated (Fig. 3C), demonstrating that *SNHG29* loss impairs leukemic propagation in vivo. These data provide an in vivo proof-of-concept for the therapeutic potential of targeting *SNHG29* in AMKL.

### SNHG29 interacts with the oncofetal RBP IGF2BP1

**Fig. 4:**
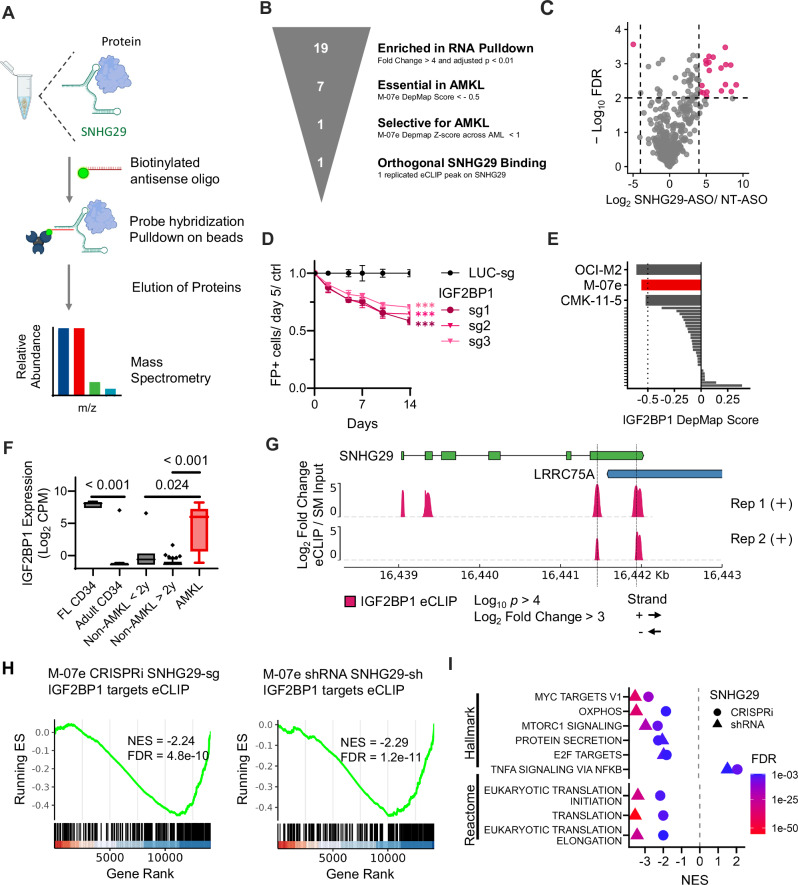
SNHG29 interacts with the oncofetal RNA-binding Protein IGF2BP1 to sustain AMKL proliferation. **A** Schematic representation of RNA pulldown strategy for identification of *SNHG29*-interacting proteins. **B** Schematic representation of our approach in determining a potentially mechanistic interaction with *SNHG29* in M-07e. **C** RNA pulldown of *SNHG29* in M-07e cells. Significantly enriched proteins (Log2 Fold Change >4, adjusted *P* < 0.01) highlighted in pink (*n* = 3 biological replicates, *t* test, BH multiple testing correction, analysis by Perseus). **D** Fluorescence-based proliferation assays in M-07e cells with CRISPR-Cas9-mediated IGF2BP1 knockout using 3 sgRNAs. Data normalized to day 0 and respective controls. (mean ± s.e.m; *P < 0.05, **P < 0.01, ***P < 0.001; two-tailed, unpaired *t* test) **E** Dependency scores (DepMap 25Q3) of IGF2BP1 across 29 AML cell lines. **F** Expression of IGF2BP1 in RNA sequencing datasets from fetal liver CD34+ cells (FL CD34, *n* = 5), peripheral blood mobilized CD34+ cells (PB CD34, *n* = 8), AMKL (*n* = 19), and non-megakaryoblastic infant (*n* = 7) and non-infant (*n* = 82) AML patient samples. Data shown are Log2 transformed TPM. Box plots show medians, boxes and whiskers according to the Tukey method. ***FDR < 0.001 (LIMMA-voom). **G** eCLIP peak data showing IGF2BP1 binding at the *SNHG29* locus in K562. Plus strand peaks with - Log10 P > 4 and Log2 fold change > 3 over size-matched input are displayed. No significant peaks were detected on the minus strand. (*n* = 2) **H** Gene Set Enrichment Analysis (GSEA) of the top 200 IGF2BP1 eCLIP targets following *SNHG29* knockdown in M-07e cells using CRISPRi with two different sgRNAs (left) or two different shRNAs (right) analyzed compared to the respective non-targeting control. Genes from RNA-seq experiments (*n* = 3) were ranked by t-statistic. NES Normalized Enrichment Score, FDR False Discovery Rate, ES Enrichment Score. **I** GSEA of Hallmark and Reactome gene sets following *SNHG29* knockdown via shRNA and CRISPRi (*n* = 3). Gene sets with the highest averaged NES between shRNA and CRISPRi conditions are shown.

To investigate the mechanism by which *SNHG29* promotes AMKL proliferation, we performed RNA pulldown assays coupled with LC-MS/MS proteomic analysis (Fig. 4A). Antisense oligos tiling the *SNHG29* locus were screened by RNase H accessibility to identify optimal capture sites (Fig. S1D). Specific enrichment of *SNHG29* was confirmed by qRT-PCR in pulldown eluates (Fig. S1C), and SDS-PAGE revealed distinct protein bands absent from controls (Fig. S1E). LC-MS/MS identified 19 high-confidence proteins enriched in *SNHG29* pulldowns (Fig. 4C, Table S1).

To prioritize functionally relevant interactors, we integrated proteomic enrichment, DepMap [45] CRISPR dependency data, and eCLIP binding profiles (Fig. 4B). Of the 19 candidates (fold change >4, FDR < 0.01), seven were essential for M-07e survival (DepMap score <–0.5, Fig. S2A). Of these, only IGF2BP1 showed selective dependency in M-07e (DepMap Z-score <–1, Fig. 4E). Most other proteins represented common essential splicing factors. Cross-referencing with eCLIP datasets [46] showed IGF2BP1 binding on *SNHG29* exons (Fig. 4G). This integrative filtering approach thus pinpointed IGF2BP1 as the principal mechanistic partner of *SNHG29* in AMKL.

### The SNHG29–IGF2BP1 interaction drives AMKL dependency

CRISPR knockout of IGF2BP1 in M-07e cells phenocopied *SNHG29* loss, causing robust growth inhibition (Fig. 4D). In DepMap [45] data, this dependency was restricted to AMKL-derived lines (M-07e, CMK-11-5, and the erythroid-like OCI-M2), whereas other AML subtypes were unaffected (Fig. 4E). eCLIP analysis [46] confirmed two IGF2BP1 binding sites on the terminal exon of *SNHG29* (Fig. 4G). Notably, *IGF2BP1* is expressed in fetal hematopoietic progenitors, silenced in adult hematopoiesis, and retained specifically in AMKL. IGF2BP1 expression is low in non-AMKL cases irrespective of age at diagnosis, indicating this association is subtype-specific rather than age-dependent (Fig. 4F). More broadly, AMKL blasts exhibited upregulation of fetal hematopoietic gene signatures compared to non-AMKL AML subtypes (Fig. S1H).

RNA-seq data revealed strong correlation between *SNHG29* expression and IGF2BP1 target genes, including *MYC* and *E2F1*, across AMKL samples (Fig. S2B), whereas *IGF2BP1* transcript levels alone did not correlate with *SNHG29*. These findings suggest that *SNHG29* modulates *IGF2BP1’s* functional output rather than its abundance.

To assess the transcriptional consequences of *SNHG29* loss, we performed RNA-seq following CRISPRi or shRNA-mediated depletion of *SNHG29*. Expression of IGF2BP1 target mRNAs (defined by eCLIP) was consistently downregulated upon *SNHG29* knockdown (Fig. 4H), as were of *MYC* and *E2F* target pathways and translational control programs (Fig. 4I). Collectively, these data suggest that *SNHG29* supports AMKL growth by stabilizing IGF2BP1-bound transcripts and maintaining downstream pathways.

### Pharmacologic inhibition of IGF2BP1 selectively impairs AMKL viability

**Fig. 5:**
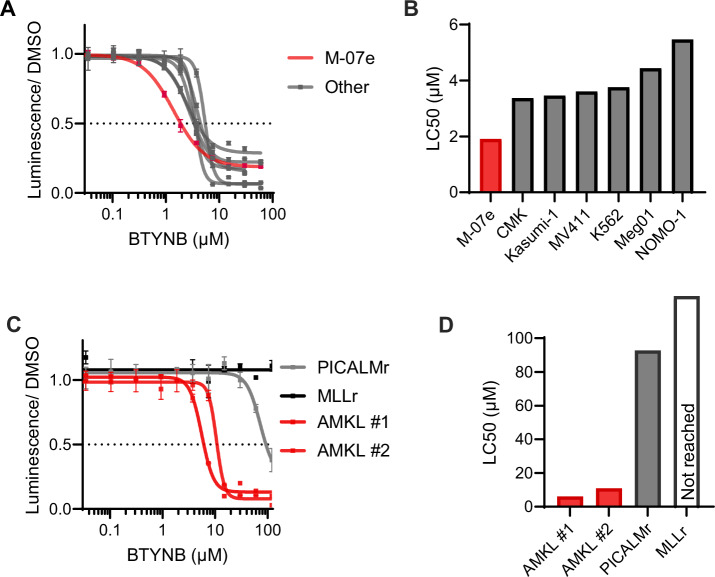
Pharmacologic inhibition of IGF2BP1 with BTYNB selectively impairs AMKL viability. **A** Dose-response curves for the AMKL cell line M-07e and non-AMKL cell lines after 72-hour treatment with the IGF2BP1 inhibitor BTYNB. Viability was measured using a luminescence-based cell viability assay and normalized to vehicle-treated controls (*n* = 3 biological replicates, mean ± s.e.m.). **B** LC₅₀ values for BTYNB in the indicated cell lines, derived from non-linear regression (*n* = 3 biological replicates). **C** Dose-response curves for AMKL and non-AMKL patient-derived blasts after a 5-day treatment with BTYNB. Viability was measured and normalized as in (**A**) (*n* = 3 biological replicates, mean ± s.e.m.). **D** Absolute LC₅₀ values for BTYNB in AMKL and non-AMKL patient-derived blasts, determined by non-linear regression (*n* = 3 biological replicates).

Given the dependency of AMKL cells on the *SNHG29*–*IGF2BP1* axis, we hypothesized that direct pharmacologic inhibition of IGF2BP1 would recapitulate the effects of *SNHG29* depletion. Treatment of AMKL cells with BTYNB, a small molecule that blocks IGF2BP1RNA binding, resulted in potent and selective cytotoxicity in M-07e cells, with an LC_50_ of 1.9 µM (Fig. 5A, B). Non-AMKL AML cell lines displayed markedly reduced sensitivity, indicating subtype-specific vulnerability.

We next tested BTYNB in patient-derived AMKL blasts, which showed pronounced and dose-dependent cytotoxicity (Fig. 5C). In contrast, non-AMKL pediatric AML samples, including a *KMT2A*-rearranged case, were resistant to treatment (Fig. 5D). These results demonstrate that AMKL cells are selectively sensitive to pharmacologic disruption of IGF2BP1, providing a proof-of-concept for targeting the *SNHG29*–IGF2BP1 axis as a therapeutic strategy in this disease.

Together, these results identify *SNHG29* as a lineage-specific dependency that sustains AMKL proliferation through its interaction with the oncofetal RBP IGF2BP1. *IGF2BP1* transcript levels did not correlate with *SNHG29* expression, yet IGF2BP1 target mRNAs were consistently downregulated upon *SNHG29* depletion, suggesting that *SNHG29* enhances IGF2BP1-mediated mRNA stabilization rather than regulating IGF2BP1 abundance. The selective activation of this developmental RNA-RBP circuit in AMKL creates a lineage-restricted vulnerability that can be therapeutically targeted. Pharmacologic inhibition of IGF2BP1 with BTYNB recapitulates the effects of *SNHG29* depletion, demonstrating the translational feasibility of disrupting lncRNA–RBP interactions in this aggressive leukemia.

These results define a developmental RNA-RBP dependency unique to AMKL and establish a mechanistic framework for understanding how oncofetal networks sustain malignant growth—setting the stage for therapeutic exploration of the *SNHG29*–IGF2BP1 axis.

## Discussion

This study identifies *SNHG29* as a previously unrecognized and essential lncRNA in AMKL, revealing a lineage-specific dependency that defines a new therapeutic vulnerability. Using an integrated strategy that combined CRISPRi screening, multiple knockdown approaches, and functional validation in vitro and in vivo, we demonstrate that *SNHG29* is indispensable for leukemic proliferation and survival. These results position *SNHG29* as a crucial regulator of AMKL maintenance and a potential therapeutic target in this high-risk leukemia with limited treatment options.

*SNHG29* depletion caused profound loss of cell viability, reduced clonogenic growth, and conferred a competitive disadvantage in PDX models, underscoring its functional importance. The specificity of this dependency for AMKL, and its absence in other AML subtypes, highlights a lineage-restricted vulnerability shaped by developmental context. Such selectivity suggests that targeting *SNHG29* or its effector pathways could impair leukemic growth without broadly affecting normal hematopoietic cells.

Mechanistically, *SNHG29* functions through direct interaction with the oncofetal RBP IGF2BP1, a post-transcriptional regulator of mRNAs encoding oncogenic drivers such as MYC and E2F1 [47–50]. Although IGF2BP1 has established roles in tumorigenesis, its contribution to pediatric AMKL has not been previously defined. Our findings reveal that *SNHG29* is required to sustain IGF2BP1 activity and stabilize its RNA targets. Loss of *SNHG29* phenocopies IGF2BP1 inhibition, leading to downregulation of IGF2BP1-bound transcripts and suppression of downstream proliferative pathways. These results uncover a previously unappreciated post-transcriptional circuit in which an lncRNA cooperates with an oncofetal RBP to maintain leukemic growth.

*SNHG29* enhances IGF2BP1 function rather than antagonizing it, consistent with prior observations that lncRNAs can act as positive regulators of IGF2BP family proteins. Similar mechanisms have been described for THOR and KB-1980E6.3, which promote IGF2BP1-mediated mRNA stabilization in solid tumors [51–53]. This interaction is specific to AMKL, representing a distinct oncofetal RNA axis reactivated in hematologic malignancy. This context-dependent interaction underscores the modularity of lncRNA function and its capacity to reinforce oncogenic pathways through protein partners selectively expressed in developmental lineages.

Our data also provide a mechanistic framework to reconcile previous reports describing *SNHG29* as a tumor suppressor in *FLT3-ITD*–positive AML, where it interacts with EP300 [54]. We propose that *SNHG29*’s function is dictated by the cellular context and availability of lineage-specific cofactors. In the oncofetal environment of AMKL, characterized by high IGF2BP1 expression, *SNHG29* is co-opted into an oncogenic role. This plasticity exemplifies how lncRNAs can adopt distinct, even opposing, functions depending on transcriptional and epigenetic context.

The identification of a selective *SNHG29*–IGF2BP1 dependency offers immediate translational potential. Direct targeting of lncRNAs remains technically challenging, but their protein effectors are more amenable to pharmacologic inhibition. We show that AMKL cells are selectively sensitive to BTYNB, a small-molecule inhibitor that disrupts IGF2BP1–RNA interactions [55]. Because BTYNB targets the RNA-binding domain of IGF2BP1, it is expected to disrupt IGF2BP1–RNA interactions broadly, including both its association with SNHG29 and with target mRNAs. Delineating the relative contribution of disrupting the SNHG29–IGF2BP1 interaction versus IGF2BP1–target mRNA binding to the antiproliferative effect will require characterization of IGF2BP1–RNA complexes under pharmacologic inhibition. Treatment with BTYNB potently reduced viability in AMKL cell lines and patient-derived blasts while having limited effects on other AML subtypes. This selective sensitivity supports a model in which AMKL cells are dependent on IGF2BP1-mediated RNA stabilization and demonstrates that pharmacologic interference with RNA–protein complexes can be therapeutically viable.

Our findings also contribute to the evolving understanding of lncRNA-mediated oncogenesis. Several pro-leukemic lncRNAs regulate gene expression at the transcriptional level [56], exemplified by HOTTIP [57], or modulate cytoplasmic signaling pathways such as HOXA10-AS [58]. *SNHG29* belongs to a distinct class of lncRNAs that act at the post-transcriptional level, directly modulating RBP activity to control mRNA fate. This mechanism highlights the functional versatility of lncRNAs as integrators of RNA metabolism and cancer signaling. Moreover, the activation of an oncofetal RBP in AMKL suggests that malignant cells can repurpose fetal regulatory networks to sustain proliferation, a concept increasingly recognized across pediatric cancers.

The role of IGF2BP1 in AMKL aligns with its role during fetal hematopoiesis and indicates that developmental gene expression programs are aberrantly retained in this leukemia. By partnering with *SNHG29*, IGF2BP1 reinstates a fetal post-transcriptional landscape that promotes leukemic maintenance. This model provides a developmental rationale for AMKL pathogenesis and reveals how fetal regulators can be activated to sustain malignancy. Understanding how these programs are re-engaged in cancer will be key to defining selective therapeutic opportunities. The context specificity of the *SNHG29*–IGF2BP1 dependency raises further questions about its regulation. Both factors are upregulated in AMKL, potentially driven by developmental transcription factors or epigenetic reprogramming unique to AMKL. Identifying these upstream regulators could explain how this oncofetal circuit is selectively activated in the megakaryoblastic lineage and may reveal additional therapeutic entry points. It will also be important to determine whether similar lncRNA–RBP partnerships operate in other pediatric or developmental cancers.

The selective vulnerability of AMKL cells to IGF2BP1 inhibition suggests that such lineage-restricted dependencies could define synthetic lethal opportunities in cancer. The developmental confinement of this axis may also reduce toxicity risks, as adult tissues largely silence both *SNHG29* and IGF2BP1. These features make the *SNHG29*–IGF2BP1 pathway particularly attractive for targeted therapy, offering a rare combination of mechanistic specificity and potential therapeutic index.

While this study establishes *SNHG29* as a key regulator of IGF2BP1 activity in AMKL, several questions remain. Structural elucidation of the *SNHG29*–IGF2BP1 interface will be important to define the molecular basis of their cooperation and could inform rational drug design. Expanded analyses across primary patient samples are needed to validate the generality of this dependency and its potential relevance in other hematologic malignancies. Finally, BTYNB establishes proof-of-concept for pharmacologic disruption of IGF2BP1–RNA interactions but will require substantial optimization and comprehensive pharmacodynamic and safety evaluation to enable clinical translation.

In summary, our work uncovers a developmental RNA circuit in which the lncRNA *SNHG29* cooperates with the oncofetal RBP IGF2BP1 to sustain leukemic gene expression programs in AMKL. This axis defines a lineage-restricted dependency that can be therapeutically exploited. By elucidating a post-transcriptional mechanism linking fetal RNA regulation to leukemic maintenance, our findings expand the paradigm of lncRNA–RBP cooperation in cancer and highlight the potential of targeting RNA-based vulnerabilities in pediatric malignancies.

## Supplementary information

Supplemental Material

Table S1

## Data Availability

Raw sequencing data for the CRISPRi screen and RNA-Seq of SNHG29 knockdown are available in GEO under accession numbers GSE326775 and GSE326774, respectively. Data on CRISPR dependencies in AML cell lines were sourced from the DepMap Project [45]. eCLIP datasets ENCSR125CLF and ENCSR975KIR were retrieved from ENCODE [59]. Gene expression data of normal hematopoietic cells and pediatric AML [6] are available at www.lncScape.de and in GEO under accession number GSE98854. This study relied on standard computational algorithms detailed in the Methods section. Any original code or further details necessary for reanalysis will be made available by the author upon reasonable request. Plasmids used in this study are available at Addgene (https://www.addgene.org/Jan-Henning_Klusmann/).
