# Supplementary material for: Lineage-restricted dependency on an oncofetal SNHG29-IGF2BP1 RNA axis in acute megakaryoblastic leukemia
Source: Leukemia. 2026 Jun 30;40(8):1778–87. doi: 10.1038/s41375-026-03040-y (PMC13421336; doi:10.1038/s41375-026-03040-y)
Supplement: Supplementary file 1 — Supplemental Material [file 41375_2026_3040_MOESM1_ESM.pdf]

Supplementary Data

Supplementary Methods

CRISPR Library Design, Cloning and Screening

Library construction involved cloning a pooled set of synthesized oligonucleotide spacers (Integrated DNA Technologies) into the SGL40C.EFS.dTomato vector (Addgene 89395) via BsmBI restriction sites. After transformation into XL1-Blue supercompetent cells (Agilent 200236) and plating, library representation was confirmed by colony counting. The screen itself was conducted in dCas9-KRAB.MeCP2-expressing cell lines. Cells were transduced with the lentiviral library at 30% efficacy and then cultured for 18 population doublings, ensuring a library representation of at least 1000-fold was maintained throughout. To prepare the library for sequencing, genomic DNA was extracted from samples at the beginning and end of the screen (JetFlex™ Genomic DNA Purification Kit, Thermo Fisher Scientific). The integrated sgRNA cassettes were then amplified by PCR (NEBNext® High-Fidelity 2x PCR Master Mix, New England Biolabs), gel-purified (GeneJET Gel Extraction Kit, Thermo Fisher Scientific), and sequenced on an Illumina HiSeq 2000 instrument. To identify AMKL-specific lncRNA dependencies, we analyzed the resulting 50 bp single-end reads from all cell lines using the MAGeCK MLE algorithm 0.5.9.5.[1]

Lentiviral Vectors

To perform targeted genetic perturbations, we utilized several lentiviral systems. Stable cell lines expressing the necessary effector proteins were generated with pLKO5d.SFFV.dCas9-KRAB.MeCP2.P2A.BSD for CRISPRi, pLKO5d.EFS.SpCas9.P2A.BSD (Addgene #57821) for knockout, or pLKO5d.SFFV.hfCas13d.p2a.BSD for RNA targeting. Individual sgRNAs, designed with the CCTop[2] platform, were delivered via the SGL40C.EFS.E2Crimson vector (Addgene #100894). For RNA interference, we designed shRNA sequences using the miR-N tool[3] and cloned them into either the SIN40C.SFFV.eGFP.miR30n (Addgene #169278) or SIN40C.SFFV.dTomato.miR30n (Addgene #169277) backbone via BsmBI digestion. The

sgRNA library for the CRISPRi screen was expressed from the SGL40C.EFS.dTomato vector (Addgene #89395). To perturb IGF2BP1, previously validated sgRNAs graciously provided by Prof. Hüttelmaier were utilized. For LRRC75A knockout, sgRNA sequences were derived from the GeCKO v2 library.[4] In all perturbation experiments, constructs targeting firefly luciferase were used as non-targeting negative controls.

Lentivirus Production

Lentiviral particles were generated via PEI-mediated co-transfection of HEK293T cells with the respective expression vector alongside packaging plasmids pMD2.G (Addgene #12259) and psPAX2 (Addgene #12260). Virus-containing supernatant was harvested and concentrated by ultracentrifugation. Target cells were then transduced by incubation with the concentrated virus in media supplemented with 5 µg/ml Polybrene (Sigma-Aldrich).

Fluorescence-based Proliferation Assay

The impact of individual gene perturbations on cell proliferation was assessed using competitive proliferation assays. Stable cell lines expressing the relevant Cas effector or wild-type cells (for RNAi) were transduced to create a mixed population of perturbed (fluorescent) and unperturbed cells. The proportion of fluorescent cells in the culture was then monitored by flow cytometry every 2-3 days for up to three weeks. The growth phenotype was quantified by normalizing the percentage of fluorescent cells at each time point against its starting value on day 0 and against a non-targeting (LUC) control.

Quantitative Real-Time PCR

Total RNA was extracted from cells between days 3 and 7 post-transduction using Quick-RNA™ Microprep or Miniprep Kits (Zymo Research) and contaminating genomic DNA was removed using on-column DNase digestion (Zymo Research) or the TURBO DNA-free™ Kit (Thermo Fisher Scientific). cDNA was synthesized using the High-Capacity cDNA Reverse Transcription Kit (Applied Biosystems). qPCR was then performed using SYBR™ Select Master Mix on a Quantstudio 5 or StepOnePlus™ Real-Time PCR cycler, and expression levels

were normalized to the housekeeping gene B2M. For cellular fractionation experiments the procedure was performed as previously described,[5] with B2M and GAPDH serving as cytoplasmic controls, and XIST and TERC as nuclear controls.

Flow Cytometry and Cell Sorting

Flow cytometry data were acquired on a CytoFLEX B4-R3-V5 or CytoFLEX S V4-B2-Y4-R3 instrument (Beckman Coulter) and analyzed using FlowJo™ v10.9 software (BD Biosciences). For cell sorting, a FACSAria™ II instrument running FACSDiva™ software (BD Biosciences) was utilized.

In Vivo Xenotransplantation Assay

To assess the *in vivo* effects of *SNHG29* knockdown, we employed a competitive xenotransplantation assay using murine xenograft models of AML according to previously established protocols.[6,7] *In vivo*-expanded PDXs were transduced with either GFP or dTomato shRNA vectors. Two experimental conditions were established to control for effects of fluorophore expression, GFP LUC-sh versus dTomato LUC-sh and GFP LUC-sh versus dTomato *SNHG29*-sh2. No randomization and no blinding was performed. The two populations were mixed at a 1:1 ratio, and one to two million total cells were administered via tail vein into 8-10 week old M-CSFh/h IL-3/GM-CSFh/h SIRPah/h TPOh/h RAG2-/- IL2Rg-/- (MISTRG) mice that had received 2.5 Gy irradiation. We monitored leukemic engraftment by analyzing peripheral blood every two weeks via flow cytometry. At humane endpoints, mice were euthanized, and cells were harvested from bone marrow and spleen for final analysis. No mice were excluded from analysis. The competitive disadvantage caused by *SNHG29* knockdown was determined by comparing the final ratio of the dTomato and GFP populations. All animal experiments were performed at the Goethe University Frankfurt animal facility under protocols approved by the Regierungspräsidium Darmstadt.

RNase H Accessibility Assay

To identify accessible regions on the *SNHG29* transcript, we designed a series of 21 nt anti-sense DNA oligonucleotides (ASOs) tiling its sequence, excluding those with likely off-target binding as predicted by BLAST. The assay was performed following a modified protocol from Neumann et. al.[8] Briefly, lysates from M-07e cells were adjusted to a final buffer composition of 60 mM NaCl, 50 mM Tris-HCl pH 8, 75 mM KCl, 3 mM $MgCl_2$, and 10 mM DTT. The lysates were incubated with 100 pmol of each ASO for 2 h at 4°C before the addition of RNase H. Following a 20-minute incubation at 37°C to induce cleavage, total RNA was isolated for qRT-PCR analysis using primers flanking each ASO target site.

RNA Sequencing

Raw sequencing data were processed using the nf-core/rnaseq pipeline 3.18[9,10] with default settings. Reads were aligned to the hg38 reference genome using STAR[11] 2.7.11b and quantified using Salmon[58] 1.10.3 against the Ensembl transcriptome version 108.[12] Batch effects from flow cell lane were corrected using Combat-seq,[13] setting the experimental condition as the variable of interest. Differential expression analysis was performed separately for CRISPRi and shRNA in R 4.4.2 using DESeq2 1.46.0.[14] To account for the large difference in knock-down efficacy between SNHG29-sh1 and -sh2 (Figure 2D), Log2 SNHG29 relative expression as determined by qRT-PCR was incorporated in the linear model. Log fold changes were shrunk using apeGLM[15] 1.28.0 according to best practice. Genes were ranked by t-statistic for GSEA. Gene sets Hallmark and Reactome were retrieved from MSigDB v2024.1.Hs.[16] Gene sets of RBP targets were derived from eCLIP analysis as described above. Gene set enrichment analysis was carried out using fgsea[17] 1.32.2 in R.

Patient RNA-Sequencing data was aligned and quantified as above. Genes with less than 10 counts in more than two thirds of samples of all molecular subtypes were filtered out. Length-corrected counts were normalized and variance stabilized using LIMMA-voom[18] 3.62.1. Pearson correlation to SNHG29 expression was calculated on Log2 transformed counts per million and multiple testing corrected using the Benjamini-Hochberg procedure. Differential

expression analysis was performed using LIMMA. To assess fetal hematopoietic gene expression, two complementary gene sets were used. A human fetal signature was derived from differentially expressed genes between human fetal liver and adult HSPCs, and a mouse fetal signature was derived from mouse fetal liver versus adult HSPCs with gene symbols converted to human orthologs. Signature enrichment scores were calculated using GSVA[19] 2.0.0.

DepMap Dependency Analysis

CRISPR dependency scores for *SNHG29* pulldown enriched proteins were retrieved from the DepMap portal (25Q3 release). Analysis included all 29 AML cell lines available in DepMap (**Table S6**). Genes were classified as essential in M-07e if their dependency score was < -0.5. To identify selective dependencies in M-07e, Z-scores were calculated by standardizing each gene's dependency score in M-07e against the mean and standard deviation across all 29 AML cell lines. Genes with a Z-score < -1 were considered selectively essential in M-07e.

CRISPR-Cas9 Indel Validation

The efficiency of CRISPR-Cas9-mediated gene editing was confirmed by analyzing insertions and deletions (indels). Genomic DNA was isolated from cells 3 days post-transduction using the Quick-DNATM Miniprep Kit (Zymo Research). A PCR amplicon of approximately 500 bp flanking the sgRNA target site was generated and subjected to Sanger sequencing. The resulting sequence trace was compared to that from wild-type cells using the TIDE online tool[20] to quantify the percentage of edited alleles.

Cells and Cell Culture

Patient-derived AML cells, previously expanded *in vivo*, were thawed and cultured for 24–48 hours prior to experiments in StemSpan SFEM (STEMCELL Technologies) supplemented with 1% penicillin/streptomycin, a cytokine cocktail (50 ng/ml SCF, 50 ng/ml FLT3L, 10 ng/ml IL6, 2.5 ng/ml IL3, 10 ng/ml TPO; Peprotech), and small molecules (750 nM SR1, 35 nM UM171; STEMCELL Technologies). At 72 hours post-transduction, cells were either harvested and sorted for subsequent xenotransplantation and colony-forming assays or continuously

cultured for *in vitro* competition assays. Human myeloid leukemia cell lines K562, THP-1, ML-2, M-07e, KASUMI-1, NOMO-1 and SKNO-1 were procured from DSMZ (Braunschweig, Germany). All cell lines were maintained as per the supplier's recommendations and were subjected to routine testing for Mycoplasma contamination. Transductions were performed with the addition of 5 µg/ml Polybrene (Sigma-Aldrich). For colony-forming assays, patient material was sorted 3 days post-transduction and plated in human methylcellulose complete medium HSC003 (R&D Systems). 50,000 cells were plated over two 60 mm dishes. Colonies were counted when they reached sufficient size (10-14 days).

In Vitro Dose-Response Assay

To assess the efficacy of BTYNB, leukemia cell lines and PDX cells were seeded in 96-well plates. The cells were treated with serial dilutions of BTYNB or a DMSO vehicle control. Cell lines were incubated for 3 days, while PDX cells were cultured for 5 days. Cell viability was subsequently measured using the CellTiter-Glo® Luminescent Cell Viability Assay (Promega) following the manufacturer's instructions. Luminescence was recorded on a GloMax® Multi+ detection system (Promega). Viability data were normalized to vehicle-treated control wells. LC_{50} values, defined as the drug concentration required to reduce cell viability to 50%, were then calculated from dose-response curves using nonlinear regression analysis in GraphPad Prism 9.

Supplementary Figures

Supplementary Figure 1

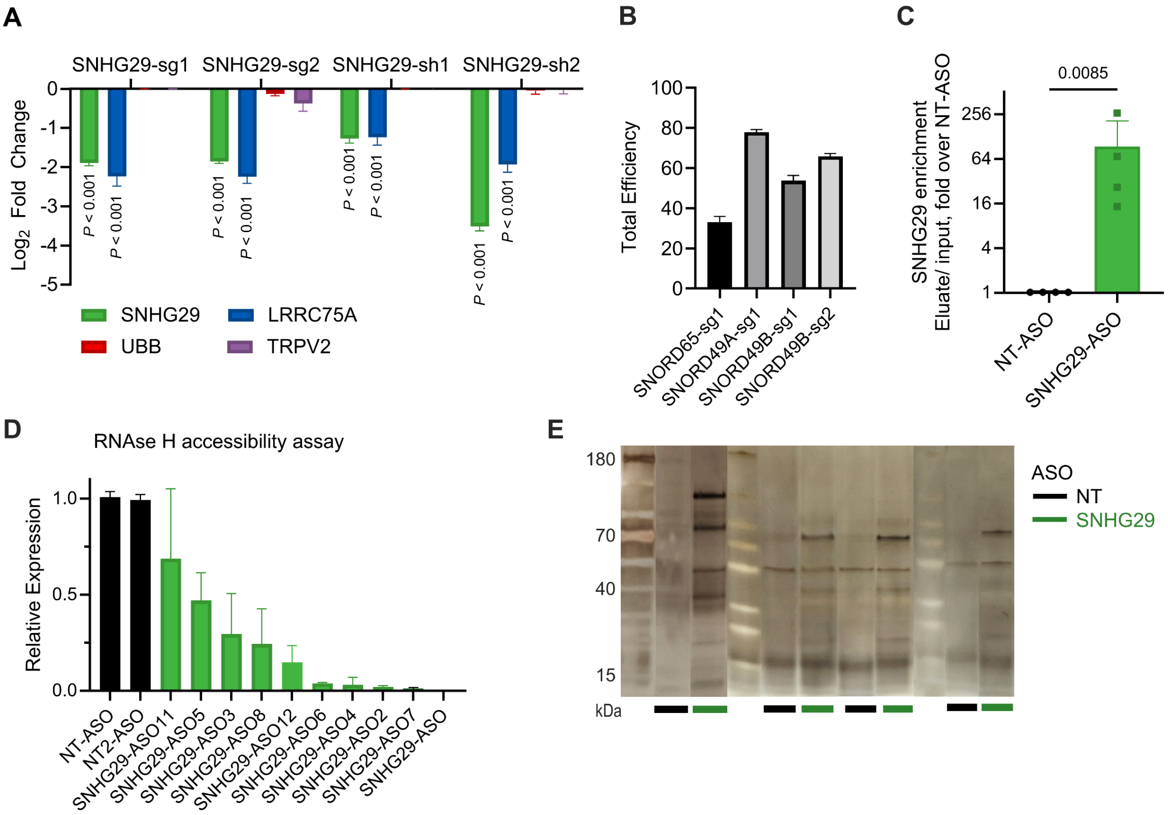

Suppl. Fig. 1 | A Expression changes of genes near the *SNHG29* locus in RNA sequencing following *SNHG29* knockdown in M-07e cells using CRISPRi with two different sgRNAs (left) or two different shRNAs (right) compared to the respective non-targeting control. Data shown are shrunken Log2 fold changes. ***FDR<0.001 (DESeq2). **B** CRISPR-Cas9 editing efficiency of indicated sgRNAs targeting SNORD65 and SNORD49A/B in M-07e Cas9 cells. Efficiency, represented as the percentage of total alleles with insertions/deletions (indels), was quantified by TIDE analysis following PCR amplification of the target sites (n=2, mean ± s.e.m.) **C** Relative *SNHG29* enrichment in the RNA affinity purification. Eluate was normalized to input and relative enrichment calculated over NT-ASO condition. (n=4 independent replicates; mean ± s.e.m.; Relative *SNHG29* enrichment compared using ratio paired t-test; **P<0.01) **D** RNase H accessibility assay using antisense DNA oligonucleotides (ASOs) tiling across the *SNHG29* transcript in M-07e cells. NT-ASO serves as a non-targeting control. Lower expression indicates higher accessibility of the target regions to RNase H-mediated degradation. Data are presented as relative expression normalized to NT-ASO control (n=2, mean ± s.e.m.). **E** Silver-stained SDS-PAGE gel of proteins eluted from the RNA pulldown assay. The gel displays proteins captured from cell lysates using either a biotinylated antisense oligonucleotide targeting *SNHG29* (ASO *SNHG29*) or a non-targeting control oligonucleotide (ASO NT). kDa: kDalton. (n=4 biological replicates).

Supplementary Figure 2

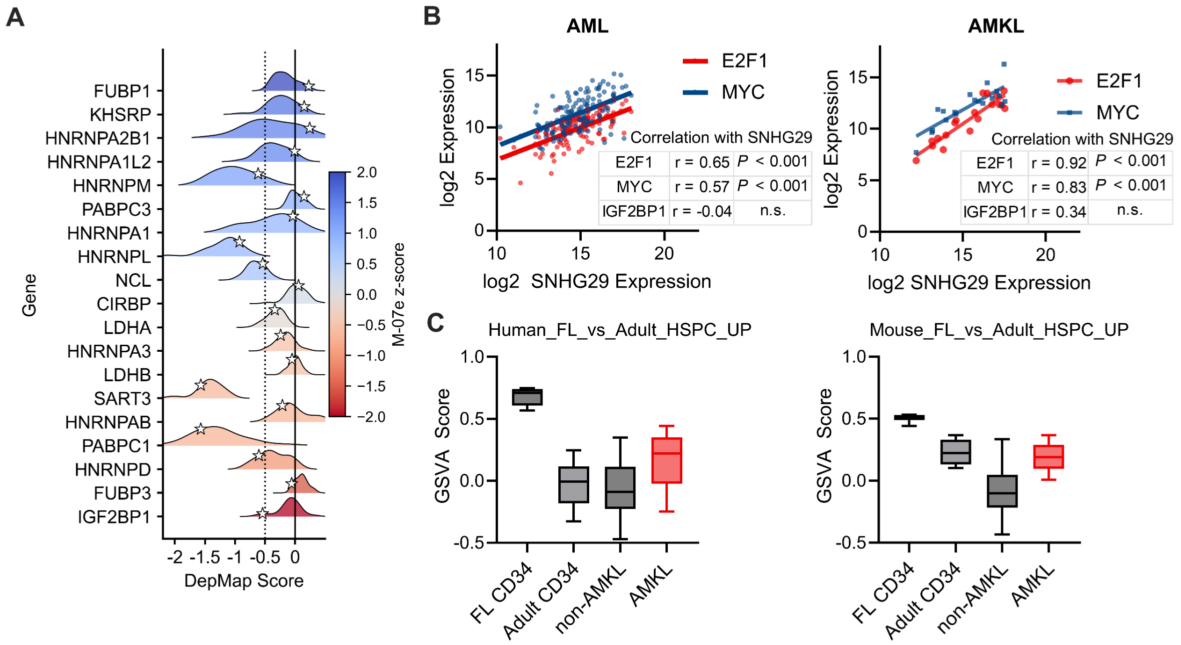

Suppl. Fig. 2 | A Dependency scores (DepMap 25Q3) of all proteins enriched in *SNHG29* pulldown. Specificity Z-scores were calculated across AML cell lines. **B** Correlation of E2F1, *MYC* and *IGF2BP1* (only statistical results shown) expression with *SNHG29* expression in non-megakaryoblastic AML (left, n=130) and AMKL (right, n=19) patient samples. Data are from RNA sequencing, Log2 transformed TPM. Pearson correlation co-efficients (r) and significance for E2F1, *MYC*, and *IGF2BP1* are indicated. ****P<0.0001; n.s., not significant. **C** Gene Set Variation Analysis (GSVA) scores of gene signatures upregulated in human (left) and mouse (right) fetal liver versus adult HSPCs in FL CD34+ (n=5), Adult CD34+ (n=8), non-AMKL (n=130) and AMKL (n=19) samples.

Supplementary Tables

Supplementary Table 1: CRISPRi lncRNA library

Supplementary Table 2: Clinical and genetic characteristics of patient cells used in this study

	Gender	Age (years)	WBC (x10³/µL)	Hemoglobin (g/dL)	BM blasts (%)	CNS	SCT	Molecular genetics	Cytogenetics	Response	Relapse
AMKL #1	m	1	40	4.59	64	no	yes	KMT2A mutation	46,XY[15]; FISH: negative (EVI1, RUNX1T1/RUNX1, MLL, CBFB, RARA)	CCR	yes
AMKL #2	f	3	n/a	n/a	n/a	n/a	yes	CKIT mutation	46,XX,add(3)(q?13), der(5) add(5)(p15) add(5)(q35) del(5) (q14q34),del(9) (q21q33), add(17) (q24)[4]/ 46,XX[11]	CCR	no
PICALMr	f	5	111	9.5	93	n/a	no	PICALM::MLLT10	t(10;11)(p12;q14)	CCR	no
MLLr	f	7	58.5	8.3	84	no	yes	KMT2A::MLLT3	46,XX, t(9;11) (p22;q23) [8]/50, i-dem, +3, +8, +18, +19[15]	CCR	no

Supplementary Table 3: sgRNA and shRNA name spacer sequences

Oligonucleotide name	System	Sequence
LUC-sg	CRISPRi	AAGAGATACGCCCTGGTTCCTGG
SNHG29-sg1	CRISPRi	GGTAGGCTCTCTAGGAATCTGGG
SNHG29-sg2	CRISPRi	AAGCCTGTCTAGAGGGCGGAGGG
SNHG29-sg3	CRISPRi	GTTATGGAGGTAGGCTCTCTAGG
Ctrl-sh	shRNA	AGGAATTATAATGCTTATCTA
SNHG29-sh1	shRNA	GAATCAGCATCATGTTTGGCA
SNHG29-sh2	shRNA	GAAAAGGCACATTGGGTATCA
LUC-Rx	CRISPR-CasRx	TGCGTCGGTAAAGGCGATGGTG
SNHG29-Rx1	CRISPR-CasRx	CCTCTGATACATAAGGCAAGCAT
SNHG29-Rx2	CRISPR-CasRx	CCAGCTCTAAAACAGCACTCTGT
SNHG29-Rx3	CRISPR-CasRx	GGCTCCAATACTCAGCTGCCAAA
LRRC75A-sg1	CRISPR-Cas9	ATACAGAACGTCGTCTAGCG
LRRC75A-sg2	CRISPR-Cas9	AGGTCAGTGGGATTCCCGAC
LRRC75A-sg3	CRISPR-Cas9	TCAATGGCAACCGGTTGACC
LRRC75A-sg4	CRISPR-Cas9	ATACAGAACGTCGTCTAGCG
SNORD65-sg1	CRISPR-Cas9	TTCACCACTACACAATCTGCCGG
SNORD49A-sg1	CRISPR-Cas9	AGACTTGACTGCAATCAGACAGG

SNORD49B-sg1	CRISPR-Cas9	TTCCTATTACAAGTATCATCAGG
SNORD49B-sg2	CRISPR-Cas9	GTCCTGATGATACTTGTAATAGG
IGF2BP1-sg1	CRISPR-Cas9	CAAGATCATCTTACAAGCGG
IGF2BP1-sg2	CRISPR-Cas9	AATGGCACCCACATACTGGG
IGF2BP1-sg3	CRISPR-Cas9	CTCGTCCGGGCAGTCCACGA

Supplementary Table 4: PCR primers

Target	Application	Direction	Sequence
SNHG29	qRT-PCR	Forward	CATGGTCCAGGAGCTGCTG
		Reverse	AGCGATACAGAACGTCGTCTAG
B2M	qRT-PCR	Forward	TCTCTCTTTCTGGCCTGGAG
		Reverse	AATGTCGGATGGATGAAACC
SNORD49A & B	TIDE PCR	Forward	GTATTGGACAGCCTGGCAGG
		Reverse	CCAGCCTCAGGGGAGTTGTA
SNORD65	TIDE PCR	Forward	GCTGTTTTAGAGCTGGCAGC
		Reverse	GTGGGAGGATTGCTTAGGCC

Supplementary Table 5: Antisense oligos

ASO name	Application	Sequence
SNHG29-ASO	RNA pulldown	mGmUmAmAmUmGmAmAmUmG-<br>mAmUmAmCmCmCmAmAmUmGmU<br>/iSp9/rArCrGrArUrC/3deSBioTEG/
NT-ASO	RNA pulldown	mGmGmAmCmGmAmUmUmCmG-<br>mAmUmCmGmAmUmAmAmUmCmU<br>/iSp9/rArCrGrArUrC/3deSBioTEG/
NT-ASO	Accessibility assay	GGACGATTCGATCGATAATCT
NT2-ASO	Accessibility assay	GCAAGGAACGTGTGAGACTA
SNHG29-ASO2	Accessibility assay	ATTCTTCACGAATTTGCAACC
SNHG29-ASO3	Accessibility assay	AATACTCAGCTGCCAAACATG
SNHG29-ASO4	Accessibility assay	TTCTCAAAACCTCATGGCAGG
SNHG29-ASO5	Accessibility assay	CAGCTCTAAAACAGCACTCTG
SNHG29-ASO6	Accessibility assay	GGAATATAACCTTCTCTTGGG
SNHG29-ASO7	Accessibility assay	AATCCAAACTGATGGCAGCTA
SNHG29-ASO8	Accessibility assay	TTGATGCCAGTTAGTTTTCAG
SNHG29-ASO11	Accessibility assay	ATATATCTCTTGGATCTGCTG
SNHG29-ASO12	Accessibility assay	CCTTTTCTAGAAAAAGTTGCC
SNHG29-ASO	Accessibility assay	GTAATGAATGATACCCAATGT

Supplementary References

1 Li W, Köster J, Xu H, Chen C-H, Xiao T, Liu JS *et al.* Quality control, modeling, and visualization of CRISPR screens with MAGeCK-VISPR. *Genome Biol* 2015; **16**: 281.

2 Stemmer M, Thumberger T, Keyer M del S, Wittbrodt J, Mateo JL. CCTop: An Intuitive, Flexible and Reliable CRISPR/Cas9 Target Prediction Tool. *PLOS ONE* 2015; **10**: e0124633.

3 Adams FF, Heckl D, Hoffmann T, Talbot SR, Kloos A, Thol F *et al.* An optimized lentiviral vector system for conditional RNAi and efficient cloning of microRNA embedded short hairpin RNA libraries. *Biomaterials* 2017; **139**: 102–115.

4 Sanjana NE, Shalem O, Zhang F. Improved vectors and genome-wide libraries for CRISPR screening. *Nat Methods* 2014; **11**: 783–784.

5 Cabianca DS, Casa V, Bodega B, Xynos A, Ginelli E, Tanaka Y *et al.* A long ncRNA links copy number variation to a polycomb/trithorax epigenetic switch in FSHD muscular dystrophy. *Cell* 2012; **149**: 819–831.

6 Bhayadia R, Krowiorz K, Haetscher N, Jammal R, Emmrich S, Obulkasim A *et al.* Endogenous Tumor Suppressor microRNA-193b: Therapeutic and Prognostic Value in Acute Myeloid Leukemia. *J Clin Oncol* 2018; **36**: 1007–1016.

7 Al-Kershi S, Bhayadia R, Ng M, Verboon L, Emmrich S, Gack L *et al.* The stem cell–specific long noncoding RNA HOXA10-AS in the pathogenesis of KMT2A-rearranged leukemia. *Blood Adv* 2019; **3**: 4252–4263.

8 Neumann P, Jaé N, Knau A, Glaser SF, Fouani Y, Rossbach O *et al.* The lncRNA GATA6-AS epigenetically regulates endothelial gene expression via interaction with LOXL2. *Nat Commun* 2018; **9**: 237.

9 Ewels PA, Peltzer A, Fillinger S, Patel H, Alneberg J, Wilm A *et al.* The nf-core framework for community-curated bioinformatics pipelines. *Nat Biotechnol* 2020; **38**: 276–278.

10 Harshil Patel, Phil Ewels, Jonathan Manning, Maxime U Garcia, Alexander Peltzer, Rickard Hammarén *et al.* nf-core/rnaseq: nf-core/rnaseq v3.18.0 - Lithium Lynx. 2024. doi:10.5281/ZENODO.1400710.

11 Dobin A, Davis CA, Schlesinger F, Drenkow J, Zaleski C, Jha S *et al.* STAR: ultrafast universal RNA-seq aligner. *Bioinforma Oxf Engl* 2013; **29**: 15–21.

12 Dyer SC, Austine-Orimoloye O, Azov AG, Barba M, Barnes I, Barrera-Enriquez VP *et al.* Ensembl 2025. *Nucleic Acids Res* 2025; **53**: D948–D957.

13 ComBat-seq: batch effect adjustment for RNA-seq count data | NAR Genomics and Bioinformatics | Oxford Academic. https://academic.oup.com/nargab/article/2/3/lqaa078/5909519 (accessed 13 Sep2023).

14 Love MI, Huber W, Anders S. Moderated estimation of fold change and dispersion for RNA-seq data with DESeq2. *Genome Biol* 2014; **15**: 550.

15 Zhu A, Ibrahim JG, Love MI. Heavy-tailed prior distributions for sequence count data: removing the noise and preserving large differences. *Bioinforma Oxf Engl* 2019; **35**: 2084–2092.

16 Liberzon A, Birger C, Thorvaldsdóttir H, Ghandi M, Mesirov JP, Tamayo P. The Molecular Signatures Database (MSigDB) hallmark gene set collection. *Cell Syst* 2015; **1**: 417–425.

17 Korotkevich G, Sukhov V, Budin N, Shpak B, Artyomov MN, Sergushichev A. Fast gene set enrichment analysis. 2021; : 060012.

18 Ritchie ME, Phipson B, Wu D, Hu Y, Law CW, Shi W *et al.* limma powers differential expression analyses for RNA-sequencing and microarray studies. *Nucleic Acids Res* 2015; **43**: e47.

19 Hänzelmann S, Castelo R, Guinney J. GSVA: gene set variation analysis for microarray and RNA-seq data. *BMC Bioinformatics* 2013; **14**: 7.

20 Brinkman EK, Chen T, Amendola M, van Steensel B. Easy quantitative assessment of genome editing by sequence trace decomposition. *Nucleic Acids Res* 2014; **42**: e168.
